# Preliminary study on the clinical significance of kinesin Kif18a in nonsmall cell lung cancer: An analysis of 100 cases

**DOI:** 10.1097/MD.0000000000019011

**Published:** 2020-01-24

**Authors:** Weifeng Guo, Huiqing Zeng, Jinyang Zheng, Yueming He, Xibin Zhuang, Jinghuang Cai, Hong Huang, Hongbo Huang, Meng Xu

**Affiliations:** a Department of Respiratory Medicine, First Hospital of Quanzhou Affiliated to Fujian Medical University, Quanzhou; b Fujian Medical University Union Hospital, Fuzhou; c Department of Respiratory Medicine, Zhongshan Hospital Affiliated to Xiamen University, Xiamen; d Department of Pathology, First Hospital of Quanzhou Affiliated to Fujian Medical University, Quanzhou, China.

**Keywords:** kinesin, Kif18A, nonsmall cell lung cancer, cancer tissue, tumor differentiation

## Abstract

The aim of this study was to investigate the expression of Kif18A in cancerous and paracancerous tissues from 100 patients with nonsmall cell lung cancer (NSCLC).

This was a prospective study of 100 patients with pathologically confirmed NSCLC (adenocarcinoma and squamous cell carcinoma [SCC], n = 50/group) that were operated at the Quanzhou First Hospital Affiliated to Fujian Medical University between June 2015 and December 2016. Kif18A protein expression in cancerous and paracancerous normal tissues was detected by western blot and immunohistochemistry.

The expression of the Kif18A protein was higher in adenocarcinoma and SCC tissues than in the corresponding paracancerous normal tissues. The expression of the Kif18A protein was higher in highly differentiated tumors, in patients with lymph node metastasis (vs no lymph node metastasis), adenocarcinoma, and in stage III NSCLC. There were no associations between Kif18A expression and age, gender, and pathologic type.

The expression of the Kif18A protein by immunohistochemistry was higher in NSCLC tissues than in normal tissues, and was associated with tumor differentiation, lymph node metastasis, and TNM staging. These results could provide a theoretical basis for novel molecular targeted therapies against NSCLC.

## 1 Introduction

Lung cancer is one of the malignant tumors with the highest incidence and mortality rates in the world: it ranks 1st among males, and 2nd among females.^[1]^ In China, its incidence rate has increased by 46.5% over the past 30 years.^[2]^ Most patients are at advanced stages at diagnosis,^[1]^ and the 5-year overall survival to lung cancer is still only about 15%.^[3,4]^ Nonsmall cell lung cancer (NSCLC) accounts for 85% of all lung cancers.^[5,6]^ The pathogenesis of lung cancer is a very complicated multistep and multistage process that involves many genes. The study of the molecular characteristics of lung cancer could help develop individualized treatment strategies and evaluate prognosis.

Kinesins were 1st discovered in 1985, and they play important roles in the regulation of microtubule stability and mitosis progression^[7]^; therefore, they are involved in the occurrence and development of malignant tumors,^[8]^ and various kinesin inhibitors are being developed.^[9]^ Kif18A is one of the most important members of the Kinesin-8 family and plays important roles in chromosome movement during mitosis.^[10–12]^ Kif18A is associated with the occurrence and development of gastric, liver, pancreatic, breast, ovarian, and head and neck cancers.^[13–17]^ There is no study specifically on the association between Kif18A and NSCLC, but a proteomics study of patients with lung cancer due to asbestos exposure showed that three protein peaks could predict the development of lung cancer with 87% sensitivity and 70% specificity in those patients; the first 2 peaks are KIF18A and KIF5A, respectively.^[18]^ Therefore, Kif18A could be a candidate gene for the development of lung cancer.

We hypothesized that Kif18A participates in NSCLC development. Therefore, the aim of this prospective study was to investigate the expression of Kif18A in 100 patients with NSCLC. The expression of the Kif18A protein in cancerous tissues and corresponding paracancerous normal tissues was measured, and the associations with clinical and pathologic data were analyzed.

## 2 Materials and methods

### 2.1 Subjects

**Figure 1:**
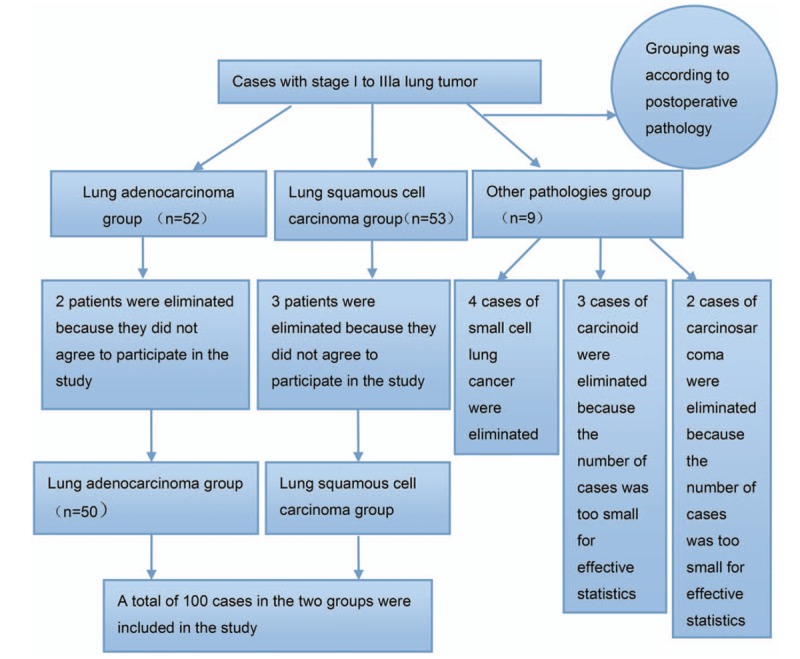
Patient flowchart.

This was a prospective study of 100 patients with pathologically confirmed NSCLC that was operated at the Quanzhou First Hospital Affiliated to Fujian Medical University between June 2015 and December 2016. Patients were consecutively enrolled until 50 patients with adenocarcinoma, and 50 patients with squamous cell carcinoma (SCC) were enrolled (Fig. 1). All patients were treatment naive (radiotherapy and chemotherapy) at the time of surgery.

This study was approved by the ethics committee of the Quanzhou First Hospital Affiliated to Fujian Medical University. The patients and their families agreed to participate in the study and signed the informed consent forms.

The inclusion criteria were: the resected tumor tissues were confirmed as NSCLC by histopathologic examination; no previous history of malignant tumors or no synchronous tumors in other organs; and the patient were treatment-naive (radiotherapy, chemotherapy, and targeted drug therapy. The exclusion criteria were: serious heart, lung, or kidney disease, or any other diseases that could affect survival.

### 2.2 Tissue sample collection

All patients were confirmed with NSCLC by histopathologic examination. All surgeons were chief physicians, and all pathologists were associate chief physicians or above.

All study specimens were obtained in the fresh state within 30 minutes after resection. The study specimens were 1.5 × 1.0 × 1.0 cm and were collected from the longitudinal section of the tumor (to avoid normal lung tissue) and from the central nonnecrotic area of the tumor. Paracancerous normal lung tissues were collected >2 cm away from the tumor margin; the absence of tumor cells was confirmed by histopathologic examination.

### 2.3 Expression of Kif18A protein by western blot

The expression of the Kif18A protein was detected by western blot. The specimens were homogenized on ice using a Potter Elvehjem glass homogenizers. The mixtures were mixed with RIPA lysis buffer and incubated on ice for 30 minutes. The lysates were collected, centrifuged at 12,000 rpm for 10 minutes at 4°C. The supernatants were used for the experiments. The protein concentrations were determined using the BCA assay. Total proteins (50 μg) were separated by sodium dodecyl sulfate-polyacrylamide gel electrophoresis and transferred on nitrocellulose membranes. The membranes were blotted with antibodies against Kif18A (dilution: 1:500; Bioss Antibodies, Inc, Woburn, MA) and β-actin (dilution: 1:1000; Sigma US). The X-ray films were photographed using a gel imaging analysis system (LY-SUPER HP CCD camera system; Chengdu Liyang Precision Machinery Co, Ltd, Chengdu, China). The gray-scale value of each band was analyzed using the Lab Works 2.0 software (Perkin-Elmer Life Sciences, Waltham, MA), with β-actin as the internal reference. The relative expression of the Kif18A protein in each group was calculated according to the gray-scale value of the Kif18A band gray-scale value to that of β-actin. Each sample of NSCLC was tested for KIF18A along with its corresponding paracancerous normal tissue sample. The expression of KIF18A in NSCLS was compared with that of the normal tissue.

### 2.4 Data collection

Data were collected from the medical charts, including sex, age, tumor differentiation, lymph node metastasis, and TNM staging.

### 2.5 Immunohistochemistry

The rabbit anti-human Kif18A antibody was used as the primary antibody (Bioss Antibodies, Inc), and was detected using a streptavidin-biotin complex (Beijing Dingguo Changesheng Biotechnology Co, Ltd, Beijing, China). All specimens were evaluated in a blind manner by 2 pathologists. If the results were inconsistent, the specimens were examined again by the 2 pathologists. Staining was evaluated by the comprehensive scoring method of staining intensity × percentage of stained cells^[19,20]^: 0 to 1 point (−); 1 to 3 points (+); 4 to 6 points (++); and 7 to 9 points (+++). The staining intensity was scored as 1 (light yellow staining), 2 (yellow-brown staining), and 3 (brown staining). The slides were observed at 40×; 5 fields were randomly selected; 200 cancer cells were counted in each field, for a total of 1000 cells. The number of positive cells was scored as: 0 for <10% positive cells, 1 point for 11% to 30% positive cells, 2 points for 31% to 50% positive cells, and 3 points for >50% positive cells. KIF18A overexpression was defined as 4 to 6 points (++); and 7 to 9 points (+++).

### 2.6 Statistical analysis

SPSS 18.0 (IBM, Armonk, NY) was used for statistical analysis. Normally distributed continuous data are expressed as mean ± standard deviation, while nonnormally distributed data were log-transformed to normalize their distribution. Categorical data are expressed as percentages and rates. The *t* test or Chi-squared test was used for comparison between the 2 groups. Two-sided *P*-values <.05 were considered statistically different.

## 3 Results

### 3.1 Characteristics of the patients

Table 1 presents the characteristics of the patients. The patients with lung SCC were 25 to 81 years of age, and 66% were males. The patients with lung adenocarcinoma were 32 to 82 years of age, and 60% were males.

**Table 1:**
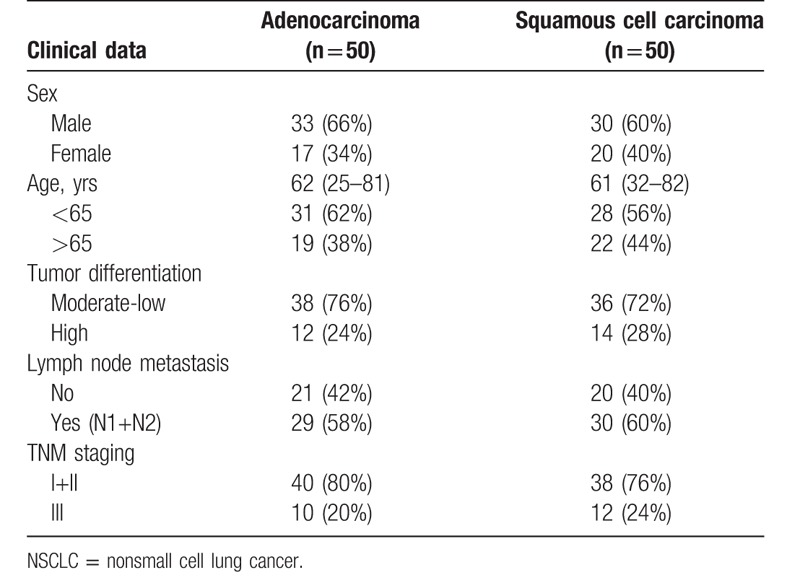
Characteristics of the patients with NSCLC.

### 3.2 Kif18A protein expression by western blot

**Figure 2:**
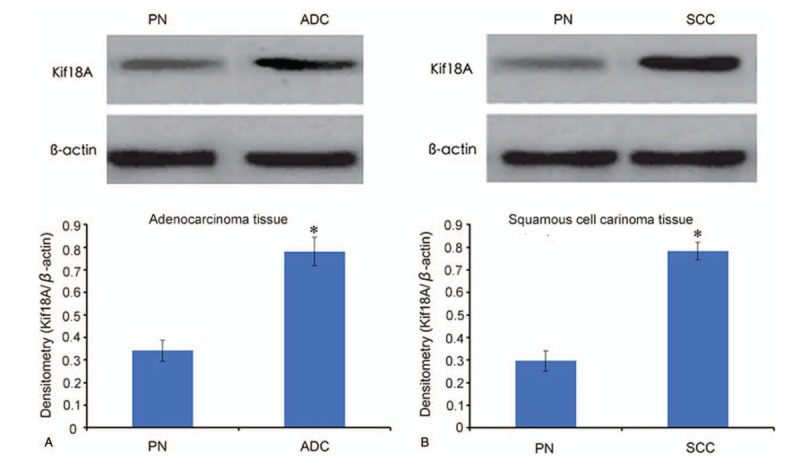
Expression of Kif18A protein by western blot in (A) lung adenocarcinoma (ADC) and (B) lung squamous cell carcinoma (SCC). The top panels present representative western blots. The bottom panel represents the average values from different samples. ^∗^*P* < .05 vs paracancerous normal (PN) tissues.

The expression of the Kif18A protein in lung adenocarcinoma tissues was higher than in the corresponding paracancerous normal tissues (*P* = .035) (Fig. 2A). The expression of the Kif18A protein in lung SCC was higher than in the corresponding paracancerous normal tissues (*P* = .042) (Fig. 2B).

### 3.3 Kif18A protein expression by immunohistochemistry

**Figure 3:**
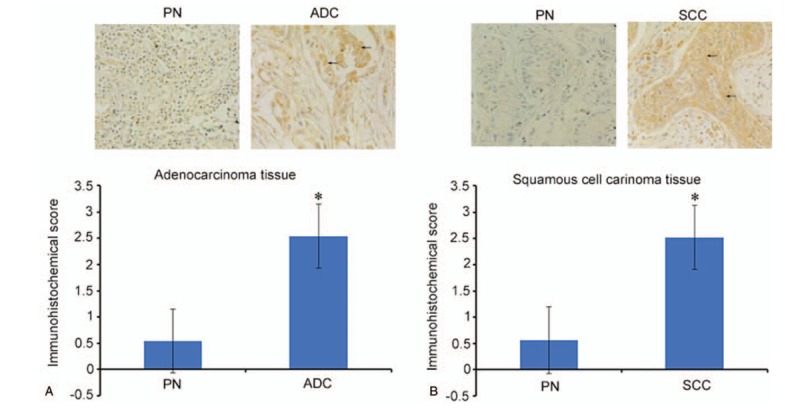
Expression of Kif18A protein by immunohistochemistry in (A) lung adenocarcinoma (ADC) (40×) and (B) lung squamous cell carcinoma (SCC) (40×). The top panels present representative immunohistochemistry images. The bottom panel represents the average values from different samples. ^∗^*P* < .05 vs paracancerous normal (PN) tissues.

The expression of the Kif18A protein in lung adenocarcinoma tissues was significantly higher than in the corresponding paracancerous normal tissues (*P* = .006) (Fig. 3A). The expression of the Kif18A protein in lung SCC was significantly higher than in the corresponding paracancerous normal tissues (*P* = .005) (Fig. 3B).

### 3.4 Association of the Kif18A protein and clinicopathologic data of patients with lung cancer

As shown in Tables 2 and 3, there were no statistical differences in the expression level of Kif18A between male and female (*P* > .05), according to age (*P* > .05), or between the 2 histologic subtypes (*P* > .05). The expression of the Kif18A protein was higher in highly differentiated tumors than in poorly/moderately differentiated tumors (adenocarcinoma: *P* = 0.032; SCC: *P* = .022). The expression of the Kif18A protein was higher in patients with lymph node metastasis than in patients without (adenocarcinoma: *P* = .041; SCC: *P* = .037). The expression of the Kif18A protein was higher in stage III NSCLC than in stage I+II NSCLC (adenocarcinoma: *P* = .029; SCC: *P* = .022). There were no associations between Kif18A expression and age, sex, and pathologic type.

**Table 2:**
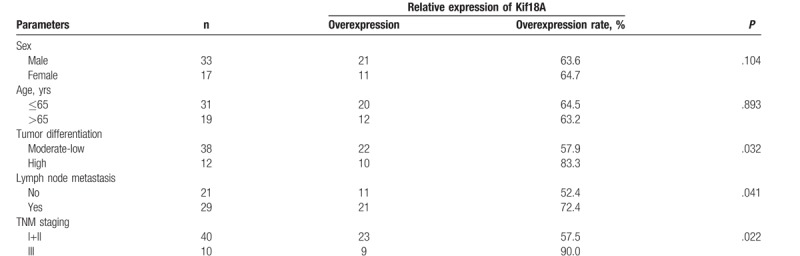
Association between Kif18A protein expression and clinical parameters in patients with lung adenocarcinoma.

**Table 3:**
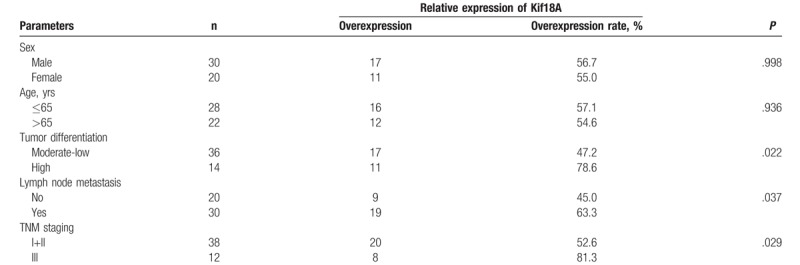
Association between Kif18A protein and clinical parameters in patients with lung squamous cell carcinoma.

## 4 Discussion

Kinesins play important roles in mitosis.^[8]^ Kif18A predicts the development of lung cancer in patients with asbestosis,^[18]^ but no data is available for NSCLC. Therefore, this study aimed to investigate the expression of Kif18A in cancerous and paracancerous tissues from 100 patients with NSCLC. The results showed that the expression of the Kif18A protein was higher by immunohistochemistry in NSCLC tissues than in normal tissues, and was associated with tumor differentiation, lymph node metastasis, and TNM staging. These results could provide a theoretical basis for novel molecular targeted therapies against NSCLC.

Kif18A plays an important role in cell mitosis. Many studies showed that Kif18A is highly expressed in breast cancer,^[21]^ rectum cancer,^[14]^ and liver cancer,^[15]^ and that it is associated with poor prognosis in those cancer types.^[14,15,21]^ On the contrary, the expression of Kif18A is low in gastric cancer, and this low expression is associated with a poor prognosis.^[22]^ Therefore, the expression pattern of Kif18A seems to vary among different cancer types, and little is known about those expression patterns. In lung cancer, Kif18A expression is associated with the development of lung cancer in patients with asbestosis.^[18]^ In the present study, the expression of the Kif18A protein in NSCLC (both SCC and adenocarcinoma) was higher than in the corresponding paracancerous normal tissues. These results indicate that Kif18A is highly expressed in NSCLC tissues, which may be related to the occurrence and development of NSCLC, as in other tumor types.^[14,15,21]^

When looking at the clinical characteristics, the results showed that sex, age, and tumor type were not associated with Kif18A protein expression, but that tumor differentiation, lymph node metastasis, and TNM staging were associated with Kif18A protein expression. In colorectal cancer, Kif18A expression is associated with tumor stage, lymph node invasion, lymphatic invasion, venous invasion, and peritoneal dissemination.^[14]^ In hepatocellular carcinoma, Kif18A expression is associated with high levels of α-fetoprotein, tumor size, TNM stage, and portal vein thrombus.^[15]^ In breast cancer, Kif18A is associated with lymph node metastasis.^[21]^ Therefore, Kif18A might play an oncogenic role in the occurrence and development of NSCLC. Nevertheless, considering that both too high^[14,15,21]^ and too low^[22]^ expression of Kif18A seem to be associated with oncogenesis, additional studies are required to determine the exact prognostic value of Kif18A.

Kinesins play important roles in the process of cell mitosis, and their abnormal expression or function can cause cells to transform into a malignant state.^[21,23–25]^ In recent years, microtubule inhibitor (MTI), kinesin spindle protein (KSP) inhibitor,^[26]^ and *S*-trityl-*l*-cysteine (STLC)^[13]^ have been successively developed and all show some activity against cancer cells.^[9]^ Therefore, kinesins could eventually be used as treatment targets.

The present study has limitations. The sample size was small and from a single center. In addition, no follow-up data was available to evaluate the prognostic value of Kif18A. Additional studies are necessary to characterize the role and value of Kif18A in NSCLC.

In conclusion, the expression of the Kif18A protein by immunohistochemistry was higher in NSCLC tissues than in normal tissues and was associated with tumor differentiation, lymph node metastasis, and TNM staging. These results could provide a theoretical basis for novel molecular targeted therapies for NSCLC.

## Author contributions

**Conceptualization:** Huiqing Zeng, Weifeng Guo, Jinyang Zheng, Xibin Zhuang, Hongbo Huang, Meng Xu.

**Data curation:** Weifeng Guo, Huiqing Zeng, Jinyang Zheng, Yueming He, Xibin Zhuang, Jinghuang Cai, Hong Huang, Hongbo Huang, Meng Xu.

**Formal analysis:** Weifeng Guo, Yueming He, Jinghuang Cai, Hong Huang.

**Investigation:** Hongbo Huang, Meng Xu.

**Methodology:** Weifeng Guo, Yueming He, Hong Huang.

**Project administration:** Jinyang Zheng, Jinghuang Cai.

**Software:** Xibin Zhuang.

**Validation:** Xibin Zhuang.

**Writing – original draft:** Weifeng Guo, Huiqing Zeng, Jinyang Zheng, Yueming He.
